# Medical News and Bulletin

**Published:** 1843-12-30

**Authors:** 


### MEDICAL NEWS AND BULLETIN.

The medical public of Paris has been greatly excited by the turn which the contest between M. Guérin and the hospital surgeons of that capital has taken. At page 92 we described, fully, the origin of this contest, which, however, it may be as well briefly to recapitulate. The hospital surgeons of Paris are all named, under the concours, after a series of very severe ordeals, by a jury appointed by the non-medical Board which governs the hospitals. This mode of nomination has been so long followed as to have become *a law* which the hospital surgeons consider it imperative on the Board to follow. The custom was, however, infringed, about twelve years ago, in favour of M. Civiale, the lithotritist, who was, at that epoch, named (without having submitted to the concours) surgeon to the Hôpital Necker. About two years ago M. Guérin, the well-known orthopædist, had several wards allotted to him at the Hôpital des Enfans, and a similar appointment has recently been conferred on M. Le Roy d'Etiolles. The hospital surgeons, in a body, have protested against the two latter nominations, and their cause has been warmly espoused by the medical press of Paris.

M. Guérin, the editor of the "Gazette Médicale," an influential weekly periodical, finding that the columns of his journal do not afford sufficient scope to his personal defence, has brought an action for libel against the editors of three other principal journals, viz., M. Vidal (editor of the "Gazette des Hôpitaux;") M. Malgaigne (editor of the "Journal de Chirurgie;") and M. Henrioz (editor of the "Experience;") from each of whom he claims twenty thousand francs damages, that is, about 800*l.*; or altogether, 2500*l.* The cause was to have come before the tribunals on the 11th of October, but, owing to the importance of the proceedings and some other motives, the trial has been deferred until the 14th of November. The action brought by M. Guérin against these gentlemen appears to be based on several grounds. M. Guérin, (as also do several other of the Parisian orthopædists,) often appears before the Academie de Médecine and other public medical bodies, as the author of papers on his special subject of practice, orthopædy — papers which, latterly, have principally been based on the cases treated at the Hôpital des Enfans. The results, thus announced, having been frequently impugned, he, some time ago, drew up a statistical report of the cases treated in his wards, and addressed it to the Hospital Board. This report, which was extremely favourable to his practice, was analysed, severely, by the medical press, and its veracity contested, especially by the three journals in question, and now M. Guérin founds his judicial attack, partly on these strictures and partly on some passages of the protestation lately made against his nomination to the Hôpital des Enfans by the Paris Hospital surgeons, among whom are the editors who have been made the objects of the action at law.

As might have been expected, M. Guérin has raised against him the entire body of Paris surgeons, who have come to the assistance of their friends in distress; and although he is supported, to a certain extent, by the Hospital Board, and by the Dean of the Faculty, who is said to be very amicably inclined towards him, it is doubtful whether he will be able to make good his case against opposition so unanimous and powerful. A protestation has been drawn up by the incriminated surgeons against the step taken by M. Guérin in prosecuting the editors of medical journals for criticisms on medical subjects, a course which, if successful, would, they say, render scientific discussion in the medical press impossible. This protestation has been signed, it is said, by nearly all the notable medical and surgical men of the day. Moreover, the official friends and nominators of M. Guérin do not seem inclined to give him their unconditional support. The Hospital Board has just named a committee to examine into the orthopædic service of the Hôpital des Enfans, which seems to imply, on the part of the Board, a doubt as to the propriety of the appointment that it had itself made, such a measure being altogether unparaleled with reference to any of the concours-appointed surgeons. Indeed, it is contrary to precedents; for, last year, on M. Bouillaud, the well-known clinical professor of La Charité, asking the Board to nominate a committee to inquire into the veracity of his statements respecting his treatment of typhus fever by repeated bleeding, which had been vehemently attacked by the medical press, the Board refused to comply; thus declining, in the one case, to adopt a measure, calculated to shield a scientific man from attack, although implored to do so by the party interested, and, in the other, spontaneously selecting the very measure which it had previously repelled. This conduct shows that in the case of M. Bouillaud the various and severe trials through which the inculpated person had passed in order to obtain his appointment were, in the eyes of the Board, sufficient guarantees of his knowledge to make the Board consider any attempt to judge between him and his antagonists nugatory and improper; while, in the case of M. Guérin—having no such guarantees of superior acquirements—the Board is alarmed, at the outcry of the profession, into doubts, both as to the talents of its nominee and the propriety of its own conduct. We are warranted in coming to this conclusion from the fact that the accusations brought against M. Bouillaud and those against M. Guérin will not bear comparison as regards their gravity, the physician to La Charité being said, by his enemies, to kill his patients "by dozens," through his *Sangrado* treatment; whereas the editor of the "Gazette Médicale" is only accused of "putting the best foot foremost," or, in other words, of presenting a more favourable statement than the facts warrant.

The quarrel which we have thus described has brought before the Paris medical public, as a necessary corollary, the interesting question, What kind of influence have "specialities" on the medical profession? and in the discussion the "specialists" are sorely beset. Indeed, they appear, as yet, to have the worst of the fight. Their position, in a medical sense, certainly is untenable. They often unjustly claim priority of invention in the departments of the profession which they exercise, and greater success than their brethren in practice. MM. Civiale and Le Roy d'Etiolles did not make their discoveries in lithotrity because they attended specially to the pathology of the urinary organs, but they became "specialists" because they had made those discoveries. Nor can they lay claim to even the greater part of the improvements which have since been made in that department of surgery, for most of them may, certainly, be ascribed to surgeons in general practice. These gentlemen are also reproached in Paris with being too much attached to their favourite modes of operating, at least of over-advocating them in their writings, and other eminent hospital surgeons are, consequently, considered by the profession to be even better authorities on lithotrity than its originators, and generally said to be equally successful in its performance. As regards orthopædy, it is justly remarked that we owe its most important operations—such as the section of the sterno-cleido-mastoideus, of the tendo Achillis, and of the muscles of the eye—to Dupuytren, Delpech, and Dieffenbach, who certainly were not "specialists;" and in all the Paris surgical wards these operations are still every day performed with as much skill and success as in any orthopædic institution. The great objection to professing and practising special branches of medicine is, in our opinion, the tendency of the habit to make mere traders of those who practise them. Praise and honour are due to the man who, having directed his attention accidentally or intentionally drawn to *one* department of medicine more than to others, and having thus acquired superior knowledge in that department, devotes himself to it, heart and soul, with a view to perfect his art for the benefit of suffering humanity, and at the same time to acquire an honorable position in the profession and society; but how few are there among special practitioners who are thus actuated.—*London Lancet.*

